# Effects of traditional Chinese exercises on insomnia after coronavirus disease 2019: A protocol of systematic review and meta-analysis

**DOI:** 10.1097/MD.0000000000031709

**Published:** 2022-11-25

**Authors:** Runtong Zhang, Wenjing Song, Luwen Zhu

**Affiliations:** a Genertec Medical Corporation Limited, Beijing, China; b Heilongjiang University of Chinese Medicine, Harbin, China; c The Second Affiliated Hospital of Heilongjiang University of Chinese Medicine, Harbin, China.

**Keywords:** coronavirus disease 2019, insomnia, meta-analysis, protocol, Traditional Chinese exercises

## Abstract

There is growing evidence that coronavirus disease 2019 (COVID-19) can trigger acute episodes of insomnia. Reports on the treatment of COVID-19-related insomnia are limited. Therefore, our study aims to perform a systematic evaluation and meta-analysis of the effects of traditional Chinese exercises (TCEs) in patients with insomnia due to COVID-19.

**Methods:**

We will search the Embase, PubMed, Cochrane Library, Web of Science, MEDLINE, Scopus, Chinese Biomedical Literature Database, Chinese National Knowledge Infrastructure Database, and Wan Fang Database from December 1, 2019 to October 2, 2022 to identify all articles on treatment of COVID-19-related insomnia using TCEs. Two researchers will screen the articles and extract the relevant information.

**Results:**

The results will provide a systematic overview of the current evidence on the use of TCE to treat patients with insomnia after COVID-19.

**Conclusions:**

The conclusions of this study will help clarify the effects of TCEs on patients with insomnia after COVID-19.

## 1. Introduction

Coronavirus disease 2019 (COVID-19), caused by a novel coronavirus, severe acute respiratory syndrome coronavirus 2,^[1]^ spread globally in 2019. On January 30, 2020, the World Health Organization Emergencies Committee declared a global health emergency based on rising case reports in China and worldwide.^[2]^ The prevalence of COVID-19 is similar to that of severe acute respiratory syndrome,^[3]^ but COVID-19 spreads more rapidly and efficiently.^[4]^ The treatment of acute infectious neocoronary pneumonia and the uncertainty of the disease affect the body and mental health.^[5]^ Surveys and meta-analyses conducted in several countries since the beginning of the pandemic^[6–8]^ reported very high and highly variable rates of insomnia (20%–35%), depression (25%–50%), and anxiety (20%–45%) in the general population,^[9,10]^ with higher rates among healthcare workers^[11,12]^ and patients with COVID-19.^[13,14]^ Clinically, insomnia is a major psychiatric disorder in adolescents. The main manifestations of insomnia include perceived sleep dissatisfaction and difficulty initiating or maintaining sleep.^[15]^ However, insomnia is a painful disorder usually associated with cognitive impairment, mood disorders, and fatigue.^[16]^ Further, a previous study showed a significantly higher rate of insomnia diagnosis in patients with COVID-19, consistent with the prediction of circadian rhythm disturbances after COVID-19.^[17]^

Traditional Chinese exercise (TCE) as a therapeutic, aerobic, and mind-body exercise is part of Chinese medicine and originated approximately 3000 years ago.^[18–20]^ The main components of TCE as a non-pharmacological intervention include Tai Chi Chuan, Ba Duan Jin, Yi Jin Jing, and 5 Animal Play. Clinical studies have shown that pulmonary rehabilitation techniques related to traditional Chinese medicine (TCM) can provide beneficial support for respiratory disorders, alleviate associated symptoms, and improve the overall quality of life. High-quality meta-analyses have shown that exercise interventions for pulmonary diseases, such as Baduanjin exercise, can improve mobility, lung function, and quality of life without adverse events and is better than conventional treatments.^[21–23]^ Moreover, studies have shown that TCEs, such as Tai Chi,^[24]^ Baduanjin,^[25]^ and Qigong,^[26]^ have significant effects on insomnia. Although sleeping pills and sedatives can relieve insomnia, their use is associated with a risk of adverse effects and drug addiction. TCEs are simple, not limited by space issues, and highly operational and implementable, which is conducive to wide dissemination. Increasing evidence has shown the effectiveness of TCE in improving the physical condition and regulating the mental health of people with mental disorders.^[27]^ However, direct evidence of the efficacy of TCE in patients with COVID-19-induced insomnia is lacking. Therefore, this study aims to perform a systematic evaluation and meta-analysis of the effects of TCEs in patients with insomnia due to COVID-19 and provide strong evidence to support their use in clinical practice for the treatment of patients with insomnia due to COVID-19.

## 2. Materials and methods

### 2.1. Protocol registration

In accordance with the guidelines, this systematic review protocol was registered at the International Prospective Register of Systematic Reviews on October 2, 2022 (registration number CRD42022366298). It will be conducted in accordance with Preferred Reporting Items for Systematic Review and Meta-Analysis Protocols 2015 statement guidelines.^[28]^

### 2.2. Inclusion criteria for study selection

#### 2.2.1. *Types of studies*.

This review will include randomized controlled trials that used TCE in patients with insomnia after COVID-19. There will be no language and publication limitation. Non-randomized controlled trials, observational studies, reviews, experimental studies, clinical case reports, and animal research will be excluded.

#### 2.2.2. *Participants*.

Patients with insomnia after COVID-19 will be included, regardless of their age, sex, educational status, or race. The diagnosis of COVID-19 will be based on the international diagnostic criteria,^[29]^ and the diagnosis of insomnia will be based on the Athens Insomnia Scale score.^[30]^

#### 2.2.3. *Types of interventions*.

All types of TCE will be included in this study. At least 1 type of TCE (i.e., Tai Chi, Wuqinxi, Baduanjin, Liuzijue, or Qigong) or modified TCE should be used to treat insomnia after COVID-19. There will be no restrictions regarding the frequency, duration, or follow-up time of treatment. The control group should be prescribed a non-TCE treatment, which can be general treatment, pharmacotherapy, or no additional intervention apart from the usual care.

### 2.3. *Outcomes*

#### 2.3.1. *Primary outcomes*.

The Insomnia Severity Index will be used to measure the severity of insomnia.^[31]^ All items will be scored on a 5-point scale, ranging from 0 (none) to 4 (very). The total score ranges from 0 to 28, and the threshold score for diagnosing insomnia disorder is ≥ 8.^[32]^

The Athens Insomnia Scale will be used to diagnose insomnia. It is a short validated questionnaire with a total score ranging from 0 to 24.^[30]^ Each item is measured on a 4-point Likert scale, with a total score ranging from 4 to 6, representing suspected insomnia symptoms. A score > 6 represents insomnia, and a score < 4 represents no insomnia.

#### 2.3.2. *Secondary outcomes*.

The Leeds Sleep Evaluation Questionnaire^[33]^ and the Pittsburgh Sleep Quality Index questionnaire will be used to measure the secondary outcomes.^[34]^

### 2.4. *Search strategy*

A literature search will be performed using 9 electronic medical databases (PubMed, Embase, Cochrane Library, Web of Science, MEDLINE, Scopus, Chinese Biomedical Literature Database, Chinese National Knowledge Infrastructure Database, and the Wan Fang Database) from December 1, 2019 to October 2, 2022 to identify all articles on treatment of COVID-19-related insomnia using TCEs. In addition, to ensure that no relevant studies are missed, we will review the bibliography of the included full-text articles. We will use a search strategy that combines subject terms with free words. The search strategy for PubMed is presented in Table 1.

**Table 1:** PubMed search strategy.

Search	Query
#1	(((((((((((((((“Sleep Initiation and Maintenance Disorders”[Mesh]) OR (Disorders of Initiating[Title/Abstract] AND Maintaining Sleep[Title/Abstract])) OR (Nonorganic Insomnia[Title/Abstract])) OR (Insomnia, Nonorganic[Title/Abstract])) OR (Primary Insomnia[Title/Abstract])) OR (Transient Insomnia[Title/Abstract])) OR (Rebound Insomnia[Title/Abstract])) OR (Sleep Initiation Dysfunction[Title/Abstract])) OR (Dysfunctions, Sleep Initiation[Title/Abstract])) OR (Sleep Initiation Dysfunctions[Title/Abstract])) OR (Insomnia Disorder[Title/Abstract])) OR (Insomnia Disorders[Title/Abstract])) OR (Insomnia[Title/Abstract])) OR (Insomnias[Title/Abstract])) OR (Chronic Insomnia[Title/Abstract])) OR (Psychophysiological Insomnia[Title/Abstract])
#2	“COVID-19”[MeSH Terms] OR “COVID-19”[Title/Abstract] OR “sars cov 2 infection”[Title/Abstract] OR “infection sars cov 2”[Title/Abstract] OR “sars cov 2 infection”[Title/Abstract] OR “sars cov 2 infections”[Title/Abstract] OR “2019 novel coronavirus disease”[Title/Abstract] OR “2019 novel coronavirus infection”[Title/Abstract] OR “2019 ncov disease”[Title/Abstract] OR “2019 ncov disease”[Title/Abstract] OR “2019 ncov diseases”[Title/Abstract] OR “disease 2019 ncov”[Title/Abstract] OR “covid 19 virus infection”[Title/Abstract] OR “covid 19 virus infection”[Title/Abstract] OR “covid 19 virus infections”[Title/Abstract] OR ((“infect”[Title/Abstract] OR “infectability”[Title/Abstract] OR “infectable”[Title/Abstract] OR “infectant”[Title/Abstract] OR “infectants”[Title/Abstract] OR “infected”[Title/Abstract] OR “infecteds”[Title/Abstract] OR “infectibility”[Title/Abstract] OR “infectible”[Title/Abstract] OR “infecting”[Title/Abstract] OR “infection s”[Title/Abstract] OR “Infections”[MeSH Terms] OR “Infections”[Title/Abstract] OR “Infection”[Title/Abstract] OR “infective”[Title/Abstract] OR “infectiveness”[Title/Abstract] OR “infectives”[Title/Abstract] OR “infectivities”[Title/Abstract] OR “infects”[Title/Abstract] OR “pathogenicity”[MeSH Subheading] OR “pathogenicity”[Title/Abstract] OR “infectivity”[Title/Abstract]) AND “covid 19 virus”[Title/Abstract]) OR “virus infection covid 19”[Title/Abstract] OR “coronavirus disease 2019”[Title/Abstract] OR “disease 2019 coronavirus”[Title/Abstract] OR “coronavirus disease 19”[Title/Abstract] OR “coronavirus disease 19”[Title/Abstract] OR “severe acute respiratory syndrome coronavirus 2 infection”[Title/Abstract] OR “sars coronavirus 2 infection”[Title/Abstract] OR “covid 19 virus disease”[Title/Abstract] OR “covid 19 virus disease”[Title/Abstract] OR ((“COVID-19”[Title/Abstract] OR “COVID-19”[MeSH Terms] OR “covid 19 vaccines”[Title/Abstract] OR “covid 19 vaccines”[MeSH Terms] OR “covid 19 serotherapy”[Title/Abstract] OR “covid 19 serotherapy”[Supplementary Concept] OR “covid 19 nucleic acid testing”[Title/Abstract] OR “covid 19 nucleic acid testing”[MeSH Terms] OR “covid 19 serological testing”[Title/Abstract] OR “covid 19 serological testing”[MeSH Terms] OR “covid 19 testing”[Title/Abstract] OR “covid 19 testing”[MeSH Terms] OR “SARS-CoV-2”[Title/Abstract] OR “SARS-CoV-2”[MeSH Terms] OR “severe acute respiratory syndrome coronavirus 2”[Title/Abstract] OR “nCoV”[Title/Abstract] OR “2019-nCoV”[Title/Abstract] OR ((“Coronavirus”[MeSH Terms] OR “Coronavirus”[Title/Abstract] OR “CoV”[Title/Abstract]) AND 2019/11/01:3000/12/31[Date - Publication])) AND “virus diseases”[Title/Abstract]) OR “disease covid 19 virus”[Title/Abstract] OR “virus disease covid 19”[Title/Abstract] OR “2019 ncov infection”[Title/Abstract] OR “2019 ncov infection”[Title/Abstract] OR “2019 ncov infections”[Title/Abstract] OR “infection 2019 ncov”[Title/Abstract] OR “COVID19”[Title/Abstract] OR “covid 19 pandemic”[Title/Abstract] OR “covid 19 pandemic”[Title/Abstract] OR “pandemic covid 19”[Title/Abstract] OR “covid 19 pandemics”[Title/Abstract]
#3	#1 AND #2
#4	((“Qigong”[MeSH] OR “Qi Gong”[Title/Abstract] OR “Chi Kung”[Title/Abstract] OR “yijinjing”[Title/Abstract] OR “baduanjin”[Title/Abstract] OR “wuqinxi”[Title/Abstract] OR “Tai Ji”[MeSH] OR “Tai-Ji”[Title/Abstract] OR “Tai Chi”[Title/Abstract] OR “Chi Tai”[Title/Abstract] OR “Tai Ji Quan”[Title/Abstract] OR “Quan Tai Ji”[Title/Abstract] OR “Ji Quan Tai”[Title/Abstract] OR “Taiji”[Title/Abstract] OR “Taijiquan”[Title/Abstract] OR “T’ai Chi”[Title/Abstract] OR “Tai Chi Chuan”[Title/Abstract] OR “traditional Chinese exercise”[Title/Abstract])
#5	#3 AND #4

COVID-19 = coronavirus disease 2019, SARS-CoV-2 = severe acute respiratory syndrome coronavirus 2.

### 2.5. Data collection and analysis

#### 2.5.1. *Selection of studies*.

**Figure 1.**
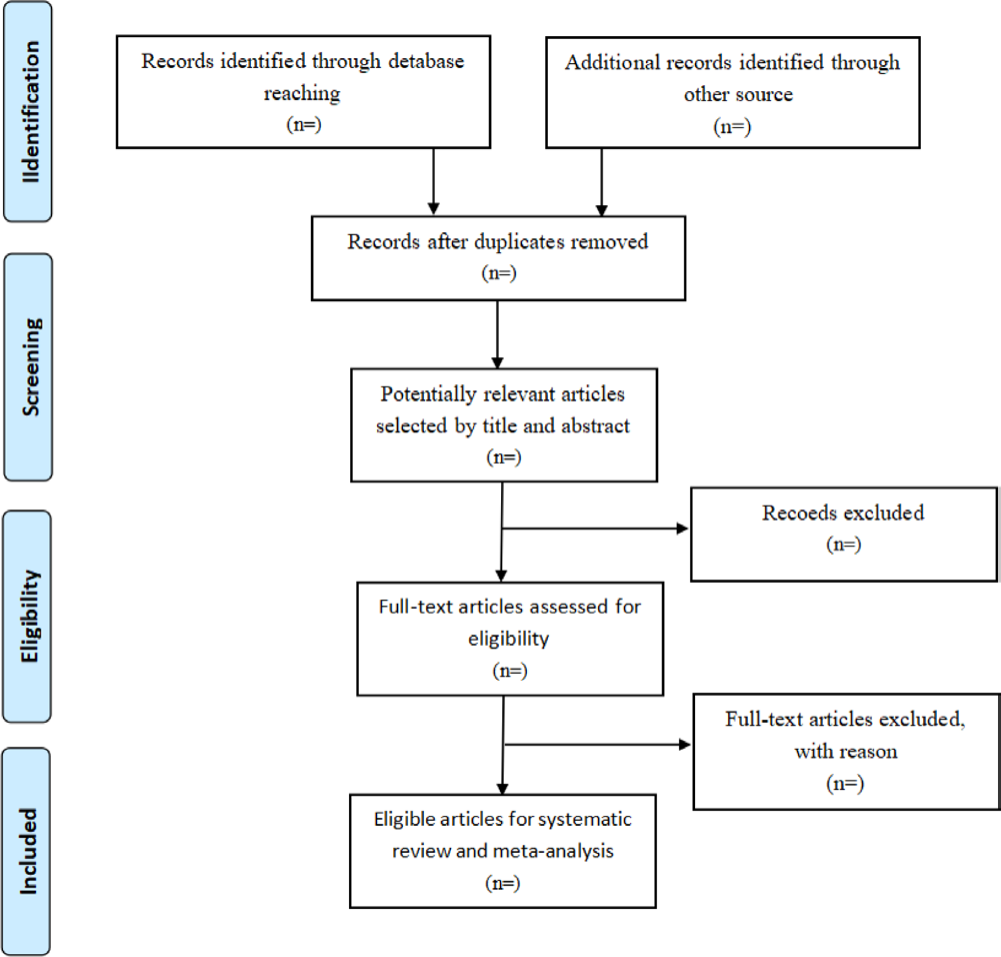
The study selection process.

Two reviewers (R-TZ and L-WZ) will independently review and screen the studies according to the inclusion and exclusion criteria of the review. EndNote V.X9 (Clarivate Analytics, Pennsylvania) will be used to manage the search results from the abovementioned databases. Two reviewers (W-JS and L-WZ) will independently identify articles by title and abstract according to the inclusion criteria, and ineligible or duplicate studies will be excluded. Then, 2 reviewers (W-JS and L-WZ) will perform full-text screening of the remaining articles, and a third reviewer (R-TZ) will resolve any disagreement between the first 2 reviewers. The screening selection process is shown in Figure 1.

#### 2.5.2. *Data collection*.

Two reviewers (R-TZ and W-JS) will independently screen the articles and extract data from the included studies. The data extraction form will include the following basic information: first author, publication year, country, study design (study type, sample size, age and sex of participants, and duration), interventions (type of TCE, duration of exercise, frequency of TCE), control (frequency and duration of treatment), outcome measures (type and number of adverse events in each group), primary outcomes, and secondary outcomes. The 2 reviewers will cross-check the completed data extraction forms. Any discrepancies will be resolved by a third reviewer (L-WZ).

#### 2.5.3. *Assessment of risk of bias*.

Two reviewers (R-TZ and L-WZ) will assess the risk of bias for each included study according to the *Cochrane Handbook for Systematic Reviews of Interventions* (The Cochrane Collaboration, 2011)^[35]^ as follows: selection bias: random sequence generation and allocation concealment; performance bias: blinding of participants and study personnel to the conditions; detection bias: blinding of outcome assessment; attrition bias: incomplete outcome data; reporting bias: selective outcome reporting of results for insomnia; and other bias. The risk of bias in each aspect will be assessed, and the results will be categorized into 3 grades ‐ low risk, unclear risk, and high risk. The 2 reviewers will cross-check the assessment results, and disagreements will be resolved by a third reviewer (W-JS).

#### 2.5.4. *Assessment of quality of evidence*.

The Grading of Recommendations Assessment, Development, and Evaluation system will be used to judge the overall quality of evidence supporting outcomes in this study. The quality of evidence will be defined as high, moderate, low, or very low.

### 2.6. *Data synthesis and analysis*

#### 2.6.1. *Data synthesis and assessment of heterogeneity*.

The RevMan.5.3 software will be used to meta-analyze the extracted data. The mean difference and 95% confidence interval will be used for continuous variables, and standardized mean difference and 95% confidence interval will be used for continuous variables if the units differ. The *Q* test and *I*^2^ statistics will be used to evaluate the heterogeneity of merged studies. *I*^2^ values of approximately 25%, 50%, and 75% indicate low, moderate, and substantial heterogeneity, respectively. When significant heterogeneity exists, a random effects model will be used; otherwise, a fixed effects model will be used.

#### 2.6.2. *Subgroup analysis*.

If the included studies show obvious clinical heterogeneity, subgroup analysis will be performed according to the clinical characteristics. We will conduct subgroup analysis according to the type of TCE, exercise time, and severity of insomnia at baseline.

#### 2.6.3. *Sensitivity analysis*.

We will use sensitivity analysis to verify that the results are robust. Methodological quality, sample size, and the effect of missing data will be included. Therefore, the impact of low-quality studies on the overall results will be evaluated.

#### 2.6.4. *Meta-regression analysis*.

If the heterogeneity cannot be resolved after subgroup analysis, we will perform meta-regression analysis on the characteristics of the article, such as country, publication year, and included population characteristics. Meta-regression will be performed if at least 10 studies will be included in the meta-analysis.^[35]^

#### 2.6.5. *Assessment of reporting biases*.

Reporting bias, including publication bias, time lag bias, duplicate publication bias, and outcome reporting bias, will be assessed using a funnel plot if the number of included studies is>10.^[36]^

#### 2.6.6. *Grading the quality of evidence*.

We will use the Grading of Recommendations Assessment, Development, and Evaluation Reliability Study system to assess the quality of the obtained evidence.

#### 2.6.7. *Dealing with missing data*.

We will attempt to contact the corresponding author for more detailed information regarding missing or unclear data. If this fails, we will analyze the available data.

### 2.7. *Dissemination*

The results of this study will be disseminated through a peer-reviewed journal.

## 3. Discussion

COVID-19, caused by severe acute respiratory syndrome coronavirus 2, continues to pose a serious public health concern.^[37]^ Patients experience various stressors during isolation, including but not limited to medication side effects, fear of serious disease consequences, fear of infecting others, and lack of timely access to correct information,^[36]^ all of which can lead to multiple psychological distress, including anxiety, depression, and insomnia.^[3,38]^ In previous meta-analyses, insomnia was positively associated with anxiety and caused fatigue in patients, which in turn was associated with several negative outcomes, such as cognitive impairment and depression.^[14,39]^ These outcomes counteract insomnia and, thus, increase the risk of insomnia.^[40]^ TCEs, such as Taijiquan and Baduanjin, which use the mind to influence bodily functions. They are very popular in complementary and alternative therapies, combining physical, emotional, spiritual, and behavioral elements through gentle movements and breathing, which can directly improve human health, focusing on the interaction between the mind and body, belonging to the mind-body therapy paradigm.^[41–46]^ Moreever, TCEs have been shown to have good efficacy in the treatment of insomnia.^[25,26]^ Our systematic review and meta-analysis may provide a reference for clinical treatment and improvement in insomnia status in patients with COVID-19.

## Author contributions

**Conceptualization:** Luwen Zhu.

**Data curation:** Wenjing Song, Luwen Zhu.

**Formal analysis:** Wenjing Song, Luwen Zhu.

**Funding acquisition:** Luwen Zhu.

**Investigation:** Runtong Zhang, Wenjing Song.

**Methodology:** Runtong Zhang, Wenjing Song.

**Project administration:** Luwen Zhu.

**Resources:** Runtong Zhang.

**Software:** Runtong Zhang.

**Supervision:** Luwen Zhu.

**Validation:** Wenjing Song.

**Visualization:** Wenjing Song.

**Writing – original draft:** Runtong Zhang, Wenjing Song.

**Writing – review & editing:** Luwen Zhu.
